# Correction: High LINC01605 expression predicts poor prognosis and promotes tumor progression via upregulation of MMP9 in bladder cancer

**DOI:** 10.1042/BSR-20180562_COR

**Published:** 2020-05-12

**Keywords:** Bladder cancer, lncRNA LINC01605, EMT, MMP9, Migration

The authors of the original article “High LINC01605 expression predicts poor prognosis and promotes tumor progression via upregulation of MMP9 in bladder cancer” (*Bioscience Reports* (2018) **38**(5); https://doi.org/10.1042/BSR20180562) would like to provide a correction to Figure 4 of their published paper. Due to a management error, an incorrect transwell migration image of sh-lnc01605-1 in T24 cells was included. The corrected Figure 4 is shown in this correction article.

**Figure 4:**
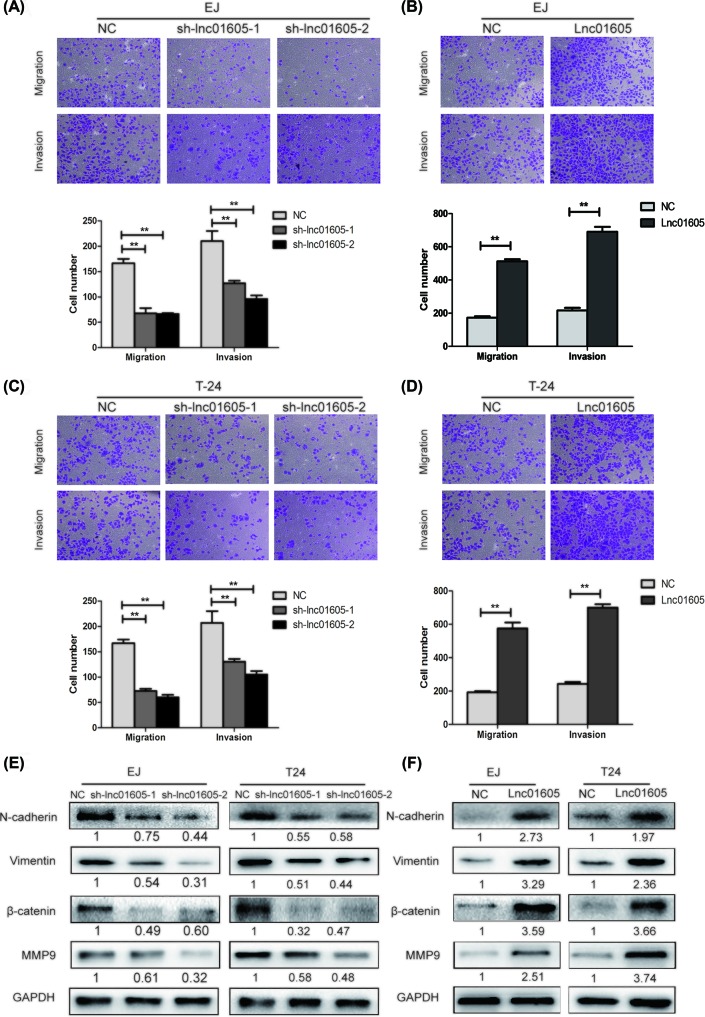
LINC01605 promotes cell migratory and invasive potential and its underlying mechanism (**A**–**D**) Transwell migration assay and Matrigel invasion assay in EJ and T24 cells with LINC01605 knockdown or overexpression. (**E,F**) N-cadherin, Vimentin, β-catenin, MMP9 protein expression levels were analyzed by Western blot in EJ and T24 cells with LINC01605 knockdown or overexpression. Data represent the mean ± S.D. from three independent experiments; **P<0.01.

The authors apologise for any inconvenience that this error has caused to the readers of their original paper.

